# Botanical Agents for the Treatment of Nonmelanoma Skin Cancer

**DOI:** 10.1155/2013/837152

**Published:** 2013-07-28

**Authors:** Jillian W. Millsop, Raja K. Sivamani, Nasim Fazel

**Affiliations:** ^1^University of Utah, School of Medicine, 30 N 1900 E, Salt Lake City, UT 84132, USA; ^2^University of California, Davis, Department of Dermatology, 3301 C Street, Sacramento, CA 95816, USA

## Abstract

Nonmelanoma skin cancers, including basal cell carcinoma and squamous cell carcinoma, are common neoplasms worldwide and are the most common cancers in the United States. Standard therapy for cutaneous neoplasms typically involves surgical removal. However, there is increasing interest in the use of topical alternatives for the prevention and treatment of nonmelanoma skin cancer, particularly superficial variants. Botanicals are compounds derived from herbs, spices, stems, roots, and other substances of plant origin and may be used in the form of dried or fresh plants, extracted plant material, or specific plant-derived chemicals. They possess multiple properties including antioxidant, anti-inflammatory, and immunomodulatory properties and are, therefore, believed to be possible chemopreventive agents or substances that may suppress or reverse the process of carcinogenesis. Here, we provide a review of botanical agents studied for the treatment and prevention of nonmelanoma skin cancers.

## 1. Introduction

Nonmelanoma skin cancer (NMSC) is common worldwide and includes basal cell carcinoma (BCC) and squamous cell carcinoma (SCC). Approximately two to three million NMSCs occur globally each year [1]. In the United States (U.S.), BCC is the most common form of malignancy [2, 3]. In 2012, two million new cases of NMSCs were estimated to occur in the U.S., with delays in treatment leading to significant morbidity [4] and deaths occurring in less than 1,000 [5]. Delay in treatment of NMSCs can lead to significant morbidity as well. Extrinsic and intrinsic risk factors contribute to skin cancer development including fair skin color, red and blond hair, and high susceptibility to sunburn [6]. Chronic sun exposure is also associated with skin cancer risk, especially for SCC [7]. Furthermore, chronically immunocompromised organ transplant patients have a higher risk of developing SCCs [8, 9].

Traditional recommendations for prevention of NMSC include generous use of sunscreens and avoidance of chronic or intense ultraviolet (UV) exposure. Conventional therapy for NMSCs involves surgical removal or directed topical therapies in the most superficial subtypes of  NMSC. However, there is increasing interest in alternative, noninvasive treatments and preventative measures in recent years, specifically in the use of naturally occurring botanicals. Botanicals are a group of compounds derived from herbs, spices, stems, roots, and other substances of plant origin and may be used in the form of dried or fresh plants or extracted plant material [10]. They possess multiple properties including antioxidant, anti-inflammatory, and immunomodulatory properties, and, therefore, they are believed to be possible chemopreventive agents or substances that may suppress or reverse the process of carcinogenesis [7]. For example, topical ingenol mebutate, extracted from *Euphorbia*, was recently approved by the U.S. Food and Drug Administration (FDA) for treatment of actinic keratosis [11]. There is perceived safety and efficacy with botanicals despite a lack of controlled clinical trials and scientific data [10]. Furthermore, botanical extracts are often distributed as dietary supplements in the United States with little regulation [12]. Therefore, there is need for controlled studies that evaluate both of their efficacy and side effects. 

 Here, we review botanical agents that have been used for the treatment and prevention of NMSCs. Botanical agents studied in humans as well as agents investigated in preclinical trials are reviewed.

## 2. Methods

We conducted a search of the PubMed and Embase databases of articles published from 1990 to 2013 to include the most recent literature. Articles containing combinations of MeSH terms “nonmelanoma skin cancer,” “basal cell carcinoma,” “squamous cell carcinoma,” “complementary therapies,” “plant preparations,” “edible plants,” “herbal medicine,” “antineoplastic agents,” “phytogenic,” and “botanical” were reviewed. Based on the initial review using these terms, botanical agents and extracts identified as having possible effects on NMSC treatment and prevention through studies, reviews, and case reports, were reviewed. The search was limited to articles in English and initially resulted in 91 articles. The article abstracts were reviewed for relevance to the subject matter of treatment and prevention of UV light-induced NMSCs, BCCs, and SCCs, using botanical agents. We examined reference lists to identify any missing articles, and a total of 74 articles were included in this review. We included only botanical agents that have been investigated in more than one study and/or have been reported in more than one case.

## 3. Botanical Agents Studied in Humans and Animals (Tables 1 and 2)

### 3.1. Ingenol Mebutate

Ingenol mebutate is an extract from the *Euphorbia peplus* (*E. peplus*) plant that has been identified for its chemotherapeutic potential, and, more recently, approved by the U.S. FDA for the treatment of actinic keratoses. Preclinical studies in murine models have shown that ingenol mebutate causes mitochondrial swelling of the dysplastic keratinocytes and apoptosis by primary necrosis [48]. In a phase I/II clinical study to determine the efficacy of the *E. peplus* sap, for which the main ingredient was ingenol mebutate, on the treatment of BCCs, SCCs, and intraepidermal carcinomas, Ramsay et al. enrolled 36 patients with a total of 48 lesions who had failed past treatments, refused surgical treatment, or were found unfit for surgical therapy due to anticoagulant use, age, site, or nature of the lesion [13]. Patients were treated once daily for three consecutive days. Complete clinical response (tumor absence after clinical examination) following treatment was 82% (*n* = 28) for BCCs, 75% (*n* = 4) for SCCs, and 94% (*n* = 16) for intraepidermal carcinomas at 1 month [13]. However, partial responses were noted in five of the BCCs treated. Although the authors note that 6 of the treated BCCs were of the nodular subtype while the rest were of the superficial subtype, they do not mention if it was the nodular subtypes that were the partially responding tumors. Also, a full histopathological examination of the treatment sites was not performed as the subjects were not suitable surgical candidates. Instead, 2–4 millimeter (mm) biopsy samplings were obtained but this was only partial sampling as the average size of the NMSC was noted to be 16 mm. The most common side effects included dry skin, erythema, and patchy moist desquamation. After a mean of 15 months for followup, the complete clinical response rates were 57% for BCCs, 50% for SCCs, and 75% for intraepidermal carcinomas [13]. For superficial carcinomas that were less than 16 mm, the complete clinical response rates were 78% (*n* = 9) for BCCs and 100% (*n* = 10) for intraepidermal carcinomas [13].

Furthermore, a phase IIa randomized, vehicle-controlled study of 60 patients evaluated the safety (primary endpoint) and efficacy (secondary endpoint) of ingenol mebutate gel for superficial basal cell carcinoma, with a lesion size of 4–15 mm. In this study, Siller et al. randomized patients to Arm A, treatment on days 1 and 2, or Arm B, treatment on days 1 and 8 [14]. Within each arm, patients were also randomized to application of ingenol mebutate gel 0.0025%, 0.01%, or 0.05%, or a matching vehicle gel [14]. No severe adverse events were reported. In four patients, severe local skin reactions occurred. The most common side effects were erythema, flaking, scaling, and dryness. The histopathological clearance rate was 71% (5/7) in the treatment group of Arm A that received 0.05% ingenol mebutate gel, and 86% (6/7) showed marked or complete clearance (50–90%) on clinical examination [14]. 

### 3.2. Hypericin

*Hypericum perforatum*, St. John's wort, contains the photoactive compound, hypericin, which is a potent photosensitizer in photodynamic therapy. It is activated by visible (400–700 nm) or UVA (320–400 nm) light [49]. Hypericin has demonstrated cytotoxic and antiproliferative properties against cancer cells. Although an early study by Alecu et al. reported that hypericin was effective for treatment of BCCs and SCCs without necrosis of the surrounding tissue when injecting hypericin solution intralesionally and then irradiating the site with visible light [15], subsequent studies did not demonstrate efficacy of hypericin in skin cancer treatment.

A prospective pilot study by Kacerovská et al. investigated the use of an extract of *H. perforatum* in combination with photodynamic therapy for BCCs, actinic keratoses, and Bowens disease (carcinoma in situ) [16]. A total of 34 patients were enrolled, 21 of which had BCCs. The extract was applied under occlusion to the skin lesions, followed two hours later by irradiation with 75 Joules per square centimeter (J/cm^2^) of red light. Patients underwent treatment for six weeks on average. They found complete clinical response for 28% of superficial BCC patients, and only partial remission was seen in nodular BCC patients [16]. By histology, a complete disappearance of cancer cells was found in 11% of the superficial BCCs [16]. All patients in the study complained of pain and burning during irradiation. The authors concluded that the results of this clinical trial showed that this combination therapy was not effective and that further studies are needed to examine the enhancement of hypericin delivery. Furthermore, a recent study by Boiy et al. demonstrated that topical hypericin with photodynamic therapy was less effective in clearance of UV light-induced tumors in mouse skin as compared to methylaminolevulinic acid with photodynamic therapy (44% clearance of skin tumors as compared to 80% clearance, resp.) [30].

### 3.3. Coffee

Coffee derives from seeds of the *Coffea* plant. Inconsistent results have been found for the association between coffee and various cancers. A prospective study by Jacobsen et al. in Norway of 13,664 men and 2,891 women demonstrated a strong inverse association between coffee drinking and NMSC [17]. Furthermore, a hospital-based case control study by Corona et al. of 166 case patients with histopathologically confirmed BCC and 158 control patients found that there was no statistically significant association between coffee consumption and BCC risk in a Mediterranean population from central-southern Italy [19].

More recently, Abel et al. performed a cross-sectional analysis of 93,676 Caucasian women in the Women's Health Initiative Observational Study to determine the relationship between consumption of coffee on a daily basis with the prevalence of NMSCs [18]. They found that women who drank caffeinated coffee daily had a 10.8% lower occurrence of NMSCs compared to women who did not drink coffee. After adjusting for lifestyle and demographic variables, they found that daily consumption of six or more cups of coffee had a 30% reduction in occurrence of NMSC [18]. Consumption of decaffeinated coffee was not related to a significant change in rate of NMSCs. The authors concluded that, in Caucasian women, daily consumption of caffeinated coffee had a dose-related association with decreased occurrence of NMSC.

### 3.4. Tea

Dried and unfermented *Camellia sinensis* leaves are often used primarily for green and black tea and have anti-inflammatory and anticarcinogenic properties. Previous mouse models demonstrated that green tea catechins or polyphenols, such as the major catechin (-)-epigallocatechin and (-)-epigallocatechin-3-gallate (EGCG), may protect against UVB radiation-induced NMSC [50, 51]. EGCG is believed to act as a scavenger of reactive oxygen species [52] and may augment the native antioxidant defense mechanisms of the cell.

A study by Lu et al. demonstrated that when hairless mice were irradiated with UVB light twice weekly for a total of 20 weeks followed three weeks later by treatment with topical caffeine or EGCG once daily for five days over 18 weeks, the number of cutaneous tumors decreased [31]. Application of caffeine reduced the number of malignant and nonmalignant skin tumors by 72% and 44%, respectively, and EGCG reduced the number of malignant and nonmalignant tumors by 66% and 55% [31]. Further immunohistochemical analysis demonstrated that EGCG and caffeine augmented apoptosis in nonmalignant cutaneous tumors and in squamous cell carcinomas, but did not affect the epidermis lacking tumor cells. Wang et al. demonstrated that oral consumption of leaf extracts from caffeinated black and green tea and decaffeinated black and green tea, similar to the composition of tea beverages for humans, decreased the risk of UVB-induced skin tumor formation in hairless mice [32].

Expanding on the animal studies, the use of green tea and its ingredients have been studied in humans. Asgari et al. performed a case-control study of 415 cases from the Kaiser Permanente Northern California database with pathology-verified SCC in 2004 to determine the association between tea consumption, containing *C. sinensis*, and SCC risk [21]. Controls were matched based on age, gender, and race and had no previous history of skin cancer. Asgari et al. demonstrated no reduction in SCC risk with regular consumption of tea, defined as consumption at least once per week (OR = 1.11, 95% confidence interval (CI): 0.81–1.54) [21]. There was no association between SCC risk and the dose or amount of tea consumed, duration or years of use, nor the “cup-years” measurement, which accounts for dose and duration simultaneously.

Rees et al. performed a case control study to determine the relationship between regular tea consumption, defined as one cup daily for one month, and the incidence of BCCs and SCCs [20]. The type of tea consumed, such as black or green tea, was not specified. Among the subjects, they had 770 people with BCC, 696 with SCC, and 715 sex- and age-matched controls. They found that regular tea consumption significantly lowered the risk of SCC (odds ratio, OR = 0.70, 95% confidence interval, CI = 0.53–0.92) [20]. This reduction was most noticeable in long-term tea drinkers and people who drank two cups daily. Similarly, the study demonstrated that tea consumption had an inverse relationship with the development of BCC risk (OR = 0.79; 95% CI = 0.63–0.98) [20]. However, this relationship was weaker with regards to SCCs. The authors report that there may be a possible effect of citrus peel used in tea associated with the decreased SCC risk, however, they did not investigate this possible association further. Furthermore, it is unclear the reason for the differences in risk for BCCs and SCCs with tea consumption.

In addition to oral consumption of tea, topical tea application was reported in a 47-year-old female with basal cell nevus syndrome. She used green tea body wraps once per month for a year without any new BCCs developing in that time. The ingredients in the gel wrap included green tea primarily, as well as other plant extracts—ginger oil, algae, calendula oil, and mustard oil [22].

### 3.5. Escharotic Agents

Black salve, a combination of botanical agents, and bloodroot are two escharotic botanical agents that have been described in isolated case reports for their use in NMSCs. One case reported a 63-year-old male with a BCC confirmed by histopathology and located below the right eye [23]. Though Mohs micrographic surgery was recommended, the patient chose to self-treat the BCC with black salve ointment containing 300 mg of bloodroot, galangal, sheep sorrel, and red clover. However, he had no improvement and, ultimately, underwent Mohs surgery. Ten years prior to developing the BCC, he had a suspected melanoma on the left naris, which he treated with black salve. Although the lesion resolved, he had extensive tissue damage at the site of application and was left with an absent left naris [23]. Another case report of an 87-year-old male with a biopsy-proven BCC treated with black salve resulted in complete loss of the nasal ala at the site of application [24]. With regards to bloodroot, a 52-year-old man with recurrent BCC on the nose treated with *Sanguinaria canadensis*, bloodroot, instead of surgery resulted in metastasis of the BCC to the distal bones and death of the patient [25]. 

### 3.6. Paclitaxel

Paclitaxel is a plant alkaloid derived from the bark of the Pacific yew tree *Taxus brevifolia*. It is known as a chemotherapy agent. Paclitaxel binds tubulin of microtubules and stabilizes the microtubule structure to block its breakdown and, therefore, inhibits cell division leading to apoptosis of the cancer cells [53, 54]. It is more commonly used to treat Kaposis sarcoma, nonsmall cell lung cancer, breast cancer, and ovarian cancer. However, there are many side effects with the intravenous administration of paclitaxel. Therefore, Paolino et al. studied the use of a topical colloid formulation for paclitaxel, paclitaxel-loaded ethosomes, a vehicle devised to decrease systemic side effects and improve patient compliance [26]. In their in vitro study, Paolino et al. demonstrated that the paclitaxel-loaded ethosomes increased the antiproliferative activity of paclitaxel in an SCC model.

Paclitaxel has also been reported in two cases for the treatment of BCC. A 60-year-old male with a sclerosing BCC on the right eyelid refractory to local therapy, surgery, radiation, and six cycles of cisplatin and capecitabine was subsequently treated with paclitaxel 175 milligrams per square meter (mg/m^2^) intravenously every 21 days for a total of 15 cycles. Complete response with elimination of the BCC was achieved after 12 cycles. The patient tolerated the treatment well and remained in complete remission documented clinically 13 months from his first cycle of treatment [27]. In another case report, a 74-year-old male with right scapular and nodal axillary relapse of BCC after surgical excision was treated with cisplatin 75 mg/m^2^ intravenously and paclitaxel 75 mg/m^2^ intravenously every 21 days. Partial response was noted after five cycles including elimination of pleural effusion, nodal and skin involvement. However, the patient died following neurotoxicity and complications of deep venous thrombosis and lung thromboembolism induced by chemotherapy [27].

### 3.7. Beta-Carotene

Beta carotene is a pigment found in a variety of plants, including carrots and richly hued vegetables. Many studies have demonstrated that a high consumption of vegetables and fruits with a high concentration of beta carotene has an inverse association with the risk of cancer [55–59]. Greenberg et al. randomly assigned 1,805 patients with a recent diagnosis of NMSC either 50 mg of beta carotene or placebo daily [28]. They followed the patients with annual skin examinations up to five years. At the five year endpoint, they found that there was no difference between the experimental and placebo groups in the rate of occurrence of the first new NMSC (relative rate, RR = 1.05; 95% CI = 0.91–1.22) [28]. In the U.S. Physicians' Health Study, a randomized, double-blind, placebo-controlled clinical trial, Frieling et al. randomized 22,071 male participants to take beta-carotene 50 mg every other day or placebo for 12 years [29]. They found that beta carotene supplementation did not affect the incidence of the first NMSC (RR = 0.98, 95% CI = 0.92–1.05), including BCCs (RR = 0.99, 95% CI = 0.92–1.06) and SCCs (RR = 0.97, 95% CI = 0.84–1.13) [29]. 

## 4. Botanical Agents Studied Only in Preclinical Studies (Table 3)

### 4.1. Curcumin

Curcumin is a spice that originates from the root of the *Curcuma longa* Linn plant and is the major contributor to the yellow pigment in turmeric and curry. It has been shown to have an anticarcinogenic effect; its proposed mechanisms of action are that it either induces apoptosis by augmenting the level of p53 [60] or it activates caspase-8, induced by the Fas receptor [61]. Topical curcumin was found to have inhibitory activity against skin tumor promotion in mice through mouse models [35–38, 58]. Furthermore, these studies demonstrated skin tumor chemoprevention through inhibition of arachidonic acid induced inflammation in vivo [37], inhibition of epidermal cyclooxygenase and lipoxygenase activity in vitro [37, 39], and suppression of oxidative stress through inhibition of leukocyte infiltration into inflammatory regions [38]. Limtrakul et al. found that Swiss albino mice given a diet containing 1% curcumin had significantly suppressed the number of dimethylbenz[*α*]anthracene (DMBA) and 12-O-tetradecanoylphorbol-13-acetate (TPA) induced skin tumors per mouse (*P* < 0.05) and the tumor volume (*P* < 0.01) as compared to mice on the standard diet [40]. Skin tumor formation was initiated by 7,12-dimethylbenz[a]anthracene (DMBA) and promoted with 12-O-tetradecanoylphorbol-13-acetate (TPA). Furthermore, in a mouse skin cancer model, Sonavane et al. found that curcumin at 15 mg applied topically had a similar effect as oral curcumin at 15 mg for inhibiting tumor growth [41]. However, no reports have been published to assess the use of curcumin in human clinical trials.

### 4.2. Genistein

Genistein is a flavonoid found in soy, Greek sage, Greek oregano, and ginkgo biloba extract and is specifically a major isoflavone in soybeans. Studies have shown that genistein have antioxidant and anticarcinogenic activity in the skin [42, 62] as well as protection against photodamage in mice [63]. Studies in mice have demonstrated that genistein inhibits skin carcinogenesis. Wei et al. demonstrated that genistein inhibited DMBA-initiated and TPA-promoted skin carcinogenesis by treating SENCAR (sensitive to carcinogenesis) mice with 10 micromoles of topical genistein for one week [43]. A later study by Wei et al. found that topical genistein also reduced ultraviolet B-induced skin tumor multiplicity and incidence in hairless mice [42]. Although there are no reported studies assessing the effect of genistein on human skin cancer development, genistein was found to inhibit UVB-induced photodamage in a small study of six men [42]. Genistein was topically applied to the six participants one hour prior to and five minutes after they were UVB irradiated. The study demonstrated that genistein applied to human skin inhibited UVB-induced erythema. More specifically, topical application of genistein prior to UVB irradiation inhibited erythema and cutaneous discomfort while application after UVB irradiation improved discomfort but had a weaker effect on erythema.

### 4.3. Grape Seed

Grape seeds contain proanthocyanidins, a group of polyphenols that have been found to possess anti-inflammatory and antioxidant properties [64, 65]. Although not extensively studied nor investigated in clinical trials, the proanthocyanidins have demonstrated possible anticarcinogenic effects to ultraviolet-induced cutaneous tumors in mice. Zhao et al. extracted proanthocyanidins in grape seeds after grape seeds were air-dried and made into a powder. They demonstrated that proanthocyanidins in grape seeds had a strong inhibitory effect for UVB-induced skin tumor development in SENCAR mice [44]. Mittal et al. found that dietary feeding of proanthocyanidins from grape seeds prevented photocarcinogenesis in hairless mice as compared to mice on the control diet [45]. Mice that consumed proanthocyanidins had reduced tumor size (29%–94%), tumor incidence (20%–95%), and tumor multiplicity (46%–95%) for UVB-induced stages of photocarcinogenesis [45]. Furthermore, they found that through in vivo and in vitro systems, photoprotection was a result of antioxidant mechanisms.

### 4.4. Lycopene

Lycopene is a carotenoid that provides the red pigmentation found in tomatoes, guava, watermelon, pink grapefruit, papaya, rosehips, and other vegetables and fruits. Though it is a phytochemical that lacks provitamin A activity, lycopene has been studied for its anticarcinogenic and antioxidant activity for breast [66], prostate [67], lung [68], and colon cancer [69]. Fazekas et al. demonstrated that topical lycopene application inhibited UVB-induced skin damage in mice [46]. Although a randomized controlled trial by Rizwan et al. found that tomato paste containing a high concentration of lycopene reduced ultraviolet-induced skin erythema and markers of ultraviolet-induced DNA damage (matrix metalloproteinase-1 and mitochondrial DNA 3895 bp) [47], there are no reports assessing lycopene effects on skin cancer in humans.

### 4.5. Silymarin

Silymarin is a flavonoid derived from *Silybum marianum*, a milk thistle plant. It is known to have antioxidant and anti-inflammatory properties [70, 71]. The primary component in silymarin is silybin, which has demonstrated to have anticarcinogenic properties for the cervix, breast, and prostate [72]. Due to its antioxidant activity, silymarin has been studied in mouse models for its effects on tumorigenesis. Katiyar et al. found that silymarin inhibited UVB-induced NMSC in hairless mice with topical silymarin in three different protocols to assess the effects of silymarin at various tumor stages [73]. Subsequently, Lahiri-Chatterjee et al. demonstrated that application of silymarin to SENCAR mice, in doses of 3, 6, and 12 mg reduced tumor incidence (by 25%, 40%, and 75%, *P* < 0.001), tumor multiplicity (76%, 84%, 97%, *P* < 0.001), and tumor volume (76%, 94%, 96%, *P* < 0.001) [74]. Silymarin has not yet been investigated in human clinical trials.

## 5. Utility against Nonmelanoma Skin Cancer

Ingenol mebutate, hypericin, coffee, tea, black salve, bloodroot, paclitaxel, and beta-carotene have been studied for their effects on NMSC in humans or have been reported to be used in humans with BCCs and SCCs. Clinical trials assessing the effectiveness of ingenol mebutate on BCCs and SCCs and case reports with patients using paclitaxel for BCCs suggest efficacy of these agents for treating NMSCs in humans. Despite favorable results, more human studies are needed to assess the chemopreventive and therapeutic effects of these botanical agents. In particular, prospective trials with efficacy as the primary outcome are still needed. Furthermore, black salve and bloodroot were reported to be more harmful than therapeutic. Participants taking beta carotene showed no difference in skin cancer rate compared to the control groups. Although clinical response has been reported using hypericin with photodynamic therapy, coffee, and tea in humans with NMSCs, there is no consensus as to the effectiveness of these agents based on the results of the studies. Finally, it is important to note that in short studies, such as the study of ingenol mebutate by Siller et al. that lasted only 12 weeks [14], histopathological examination for clearance is more appropriate rather than clinical observation for clearance. In this regard, this study was a phase IIa study with the intent to assess side effects, and the primary intent was not to assess treatment efficacy. Topical ingenol appears to be well-tolerated overall. Although it is tempting to speculate that application on sequential days may be better than spacing the applications by a week, a study specifically designed to assess efficacy is awaited before any conclusions regarding the treatment of superficial BCCs can be drawn. 

Although histopathological evaluation of clearance is the gold standard, future studies should consider additional incorporation of confocal microscopy. Confocal microscopy may have a role in noninvasively assessing superficial BCC recurrence [75–77]. Further studies will be needed to assess its efficacy in delineating recurrences in studies with botanical agents that may have a significant component of inflammation in addition to wound healing.

Five botanical agents appear to have potential for anticarcinogenesis based on preclinical studies. Curcumin, genistein, grape seed proanthocyanidin, and silymarin have demonstrated reduction in UV-induced skin cancers in mice, and lycopene has been shown to prevent photodamage in mice and humans. However, one botanical agent that has been studied for its effects on NMSCs, beta carotene, does not appear to be effective against skin cancer. Curcumin, genistein, grape seed, lycopene, and silymarin have not yet been studied in humans for the treatment or prevention of NMSC. 

## 6. Role of Lipophilicity and Hydrophilicity

An important characteristic that is highly important for topical delivery of botanical agents is their hydrophilicity and lipophilicity. Phytochemical subgroups that tend to be more hydrophilic, such as polyphenol, may not penetrate the stratum corneum well. This may explain why topical polyphenols do not seem to be as efficacious as oral/systemic delivery. On the other hand, topical delivery of more lipophilic phytochemicals, such as ingenol or silymarin, will be able to penetrate the stratum corneum more effectively. One option in conducting studies with agents that have a lower partition coefficient will be to facilitate their transit through the stratum corneum through penetration enhancers such as liposomes, iontophoresis, sonophoresis, or microneedle technology. Our suggestion for future evaluation of topical botanicals is that investigators take the octanol-water partition coefficient or the mode of extraction of the extract into account in designing future studies. For example, a nonaqueous extract or a phytochemical with a high partition coefficient may not require a penetration enhancer; on the other hand, it would be prudent to consider the use of penetration enhancers when studying aqueous extracts or phytochemicals with low partition coefficients. 

## 7. Conclusions

 In vitro and in vivo models have been developed with the hypothesis that natural products rich in complex chemicals that have been shown to induce apoptosis in animal models of chemoprevention may therefore possess the ability to arrest the cell cycle at different stages and, therefore, control the proliferation of cancer cells. Botanical agents have been studied, and some have been reported to have favorable effects against NMSC. 

In this review, we have included a variety of botanical agents that have been studied or reported for the treatment of NMSCs. A limitation of this review is that we did not include botanical agents that were reported in a single case or a single preclinical study. However, we have provided an up-to-date and concise review of botanical agents used in NMSC that is currently found in the literature. Although the limited preclinical data and clinical data available in the literature demonstrate favorable effects of some botanical agents on NMSC, for now, surgical therapy is the preferred mode of treatment for high-risk NMSCs. Botanical agents may have a preventive role in the development of cutaneous malignancies and a potentially therapeutic role for carefully chosen localized NMSCs of an appropriate subtype. The topical use of some botanical extracts and phytochemicals in the treatment of NMSC is promising. However, more studies, both preclinical and clinical, are needed to make more definitive conclusions regarding safety and efficacy.

## Figures and Tables

**Table 1:** Summary of the effects of botanical agents studied in humans.

Botanical agent	Source	Efficacy	Histopathological assessment of efficacy	References
Ingenol mebutate	*Euphorbia peplus *	Demonstrated clinical response (tumor clearance) for BCCs and SCCs.	No	[13, 14]

Hypericin	*Hypericum perforatum *	Reported clinical response for BCCs and SCCs to hypericin and photodynamic therapy in one study [15], but found the combination to be less effective in another human study [16].	Yes	[15, 16]

Coffee	*Coffea* plant	Coffee consumption was related to decreased prevalence of NMSC in two studies [17, 18]. No association between coffee consumption and NMSC in one study [19].	No	[17–19]

Tea	*Camellia sinensis *	Inconsistent results in human studies. Regular tea consumption was associated with reduced risk of SCC and BCC incidence in one study [20], but no reduction in SCC risk in another study [21]. Case reported efficacy for basal cell nevus syndrome with green tea body wrap [22].	No	[20–22]

Escharotic botanical agents	Black salve (containing bloodroot, galangal, sheep sorrel, and red clover) Bloodroot (*Sanguinaria canadensis*)	Cases reported no improvement of BCC and extensive skin necrosis with black salve application [23, 24]. Case reported metastasis of BCC with bloodroot [25].	No	[23–25]

Paclitaxel	*Taxus brevifolia *	In vitro study demonstrated topical paclitaxel increased antiproliferative activity in a squamous cell carcinoma model [26]. Demonstrated clinical response to recurrent BCC cases [27].	No	[26, 27]

Beta-carotene	*Various plants with rich hues *	No efficacy for NMSCs.	No	[28, 29]

**Table 2:** Summary of the effects of botanical agents studied in mice that have also been studied in humans.

Botanical agent	Source	Efficacy	References
Hypericin	*Hypericum perforatum *	The combination of hypericin and photodynamic therapy had poor efficacy in one mouse model [30].	[30]

Tea	*Camellia sinensis *	Animal studies demonstrate catechins have antitumor effects in mice [31–34].	[31–34]

**Table 3:** Summary of the effects of botanical agents investigated only in preclinical studies.

Botanical agent	Source	Efficacy	References
Curcumin	*Curcuma longa* Linn	Curcumin inhibits skin tumor carcinogenesis in mice.	[35–41]

Genistein	Soy, Greek sage, Greek oregano, and gingko biloba extract	Genistein inhibits skin tumor carcinogenesis in mice.	[42, 43]

Proanthocyanidin	Grape seed	Grape seed proanthocyanidins demonstrated anticarcinogenic effects against ultraviolet B-induced skin tumors in mice.	[44, 45]

Lycopene	Various plants with red pigment	Lycopene prevents photodamage in mice and humans, suggesting potential for possible prevention of NMSC.	[46, 47]
